# Perceived neighborhood environment walkability and health-related quality of life among predominantly Black and Latino adults in New York City

**DOI:** 10.1186/s12889-022-14973-1

**Published:** 2023-01-18

**Authors:** Jiaqi Zhu, Hanish Kodali, Katarzyna E Wyka, Terry T.-K. Huang

**Affiliations:** Center for Systems and Community Design, Graduate School of Public Health & Health Policy, City University of New York (CUNY), 55 West 125th Street, New York, NY 10027 USA

**Keywords:** Built environment, Walkability, Neighborhood satisfaction, Quality of life, African American, Latino, Community health

## Abstract

**Background:**

Measures of the built environment such as neighborhood walkability have been associated with health behaviors such as physical activity, the lack of which in turn may contribute to the development of diseases such as obesity, diabetes, cardiovascular disease, and cancer. However, limited research has examined these measures in association with health-related quality of life (HR-QoL), particularly in minoritized populations. We examined the relationship between perceived neighborhood environment and HR-QoL in a sample of mostly Black and Latino residents in New York City (NYC).

**Methods:**

This study utilized the baseline survey data from the Physical Activity and Redesigned Community Spaces (PARCS) Study among 1252 residents [34.6% Black, 54.1% Latino, 80.1% female, mean(±SD) age = 38.8 ± 12.5) in 54 park neighborhoods in NYC. Perceived built environment was measured using Neighborhood Environment and Walkability Survey, and mental and physical HR-QoL was estimated using Short Form (SF)-12. Using factor analysis, we identified two subscales of neighborhood walkability: enablers (e.g., trails, sidewalks, esthetics) vs. barriers (e.g., high crime and traffic). In addition, we included a third subscale on neighborhood satisfaction. Generalized Estimating Equation models adjusted for demographics and BMI and accounted for the clustering effect within neighborhood. Multiple imputation was used to account for missing data.

**Results:**

Mental HR-QoL was associated with barriers of walkability (β ± SE = − 1.63 ± 0.55, *p* < 0.01) and neighborhood satisfaction (β ± SE = 1.55 ± 0.66, *p* = 0.02), after adjusting for covariates. Physical HR-QoL was associated with only barriers of walkability (β ± SE = − 1.13 ± 0.57, *p* < 0.05).

**Conclusions:**

Among NYC residents living in minoritized neighborhoods, mitigating negative aspects of the neighborhood environment may be more crucial than adding positive features in terms of HR-QoL. Our study points to the need to investigate further the role of the built environment in urban, minoritized communities.

## Introduction

The built environment has been increasingly recognized as an important dimension in the framework of social determinants of health [1, 2]. There is a growing body of literature linking the built environment to physical and mental health [3–9]. For example, the design of urban environments, such as neighborhood walkability, transportation, food density, parks and recreational facilities, and aesthetics, has been associated with health behaviors such as diet [10] and physical activity [11]. These health behaviors, in turn, contribute to the development of diseases such as obesity, type 2 diabetes, cardiovascular disease, and cancer [12–15].

While objective physical attributes of the built environment are important (e.g., concrete amount of green space), perceptions of the environment, as proxies of how people experience their environment, may be just as critical. For example, built environment attributes have been found to have an indirect effect through the perception of the built environment on moderate-to-vigorous physical activity [16]. Indeed, perceptions of the built environment, such as the sense of satisfaction with one’s neighborhood, has been shown to mediate the relationship between objective measures of the environment and health [17, 18]. The perceived physical environment has also been found to be positively associated with physical health outcomes such as obesity [19] and mental health outcomes such as depression [20–22].

While the conventional biomedical model emphasizes disease outcomes, quality of life, as measured by various validated survey instruments, is increasingly seen as an important health outcome in its own right [23]. Research on the relationship between the built environment and measures of health-related quality of life (HR-QoL) is limited but emerging [3, 8, 24–28]. In one study, land-use heterogeneity and housing density were found to be associated with HR-QoL [28]. Perceptions of the built environment, such as perceived street noise and traffic safety, have also been related to mental and physical health components of quality of life [3, 27]. Furthermore, research has shown a positive correlation between neighborhood walkability and HR-QoL in Australia and China [26, 27]. Increased perceived diversity, safety and esthetics were found to be associated with higher physical and mental HR-QoL [27]. However, to our knowledge, little is known about these relationships in minoritized populations in the United States.

To bridge this gap, we first tested the psychometric properties of the Neighborhood Environment and Walkability Survey (NEWS) in New York City (NYC), recognizing its unique built environment compared to other major American cities. Subsequently, we examined the association between perceptions of the neighborhood environment and HR-QoL in a sample of mostly Black and Latino residents in NYC. We hypothesized that positive perceptions of the neighborhood environment would be associated with higher physical and mental components of HR-QoL.

## Method

### Study design

This study utilized the baseline adult survey data from the Physical Activity and Redesigned Community Spaces (PARCS) Study, a natural experiment evaluation on the impact of citywide park redesign and renovation on health and wellbeing [29]. The baseline survey data were collected from Summer 2016 – Spring 2018 (except during winter months) in 54 park neighborhoods throughout the five boroughs of NYC. A convenience sample of adult residents was invited to participate in the study survey.

#### Park neighborhood selection

To be included in the study, NYC Parks and Recreation identified parks in neighborhoods which met two of three selection criteria: high poverty (≥ 20% population below poverty line), high population growth (≥25% growth during 2000–2010) or high population density (≥ 110 people/acre). For the purpose of the PARCS evaluation study, study park buffer zones were defined as the area within a 0.30-mile radius from the perimeter of each park.

#### Sample selection

To meet the PARCS Study eligibility criteria, participants needed to live within the designated 0.3-mile buffer zone. Eligible participants were adults ≥18 years of age who owned a smart phone, spoke English, Spanish or Chinese (Mandarin or Cantonese), did not have any mobility issues and were intending to stay in the neighborhood for at least 4 years (due to the longitudinal nature of the parent study). This paper included observations from 1252 participants who provided any data on the relevant survey items described below.

### Measures

#### Health-related quality of life (HR-QoL)

HR-QoL is a multidimensional concept that includes individuals’ perception of their physical and mental functioning, limitations due to physical or mental health problems, bodily pain, vitality, general health, and social functioning [30]. We used the well-validated Short-Form 12 (SF-12) survey [31] in this study. Eight domains were covered by the SF-12 survey questions including 1) limitations in physical activities because of health problems 2) limitations in social activities because of physical or emotional problems 3) limitations in usual role activities because of physical health problems 4) bodily pain 5) general mental health 6) limitations in usual role activities because of emotional problems 7) vitality 8) general health perceptions. For the purpose of our analysis, we used the composite scores on mental health and physical health as our two primary outcome variables, using the standard scoring protocol for SF-12 [32]. The higher the score, the better the self-reported HR-QoL in mental health and physical health (range 0–100).

#### Neighborhood Environment and Walkability Survey (NEWS)

NEWS was developed in 2002 to measure resident perceptions of neighborhood characteristics [33], including residential density, land use mix, street connectivity, infrastructure for walking/cycling, neighborhood aesthetics, traffic and crime safety, and neighborhood satisfaction [33]. This survey we used included: places for walking and cycling (5 items), neighborhood surroundings (6 items), safety from traffic (8 items), safety from crime (6 items), and neighborhood satisfaction (18 items) [34]. Items for the first four original subscales all had four response options (1 = strongly disagree, 2 = somewhat disagree, 3 = somewhat agree, 4 = strongly agree) while items in the neighborhood satisfaction subscale had five response options (1 = strongly dissatisfied, 2 = somewhat dissatisfied, 3 = neither satisfied nor dissatisfied, 4 = somewhat satisfied, 5 = strongly satisfied). Items were coded such that the higher the subscale score, the more positive the attitude regarding the construct.

#### Covariates

Demographic variables included age, gender (female vs. male as reference group), ethnicity (Black, Other vs. Latino as reference group), BMI (overweight 25–29 kg/m^2^, obese > = 30 kg/m^2^ vs. under and normal < 25 kg/m^2^ as reference group,), annual household income (< $20,000 vs. ≥ $20,000 as reference group), education (high school graduate, some college or higher vs. less than high school as reference group), employment (self-employed, homemaker, student, retired, unable to work, unemployed for 1 year or more, unemployed for less than 1 year, don’t know/not sure vs. employed as reference group), and marital status (divorced, widowed, separated, never married, a member of an unmarried couple living together vs. married as reference group).

### Statistical analyses

NEWS was originally developed in Seattle and cross-validated in Baltimore [34]. Factor analysis has been used to adapt to countries other than the United States, such as Australia and Western Europe [35]. Due to the unique neighborhood characteristics of NYC, we first tested the psychometric properties of NEWS using exploratory factor analysis (EFA) with oblimin rotation [36]. Items with factor loading < 0.40 were dropped in subsequent analysis [37]. We used Cronbach’s alpha [38] was used to assess the internal consistency of the newly constituted subscales based on the factor analysis, considering Cronbach’s alpha > 0.7 as good and acceptable. Spearman correlation tests were performed between NEWS subscales and HR-QoL components.

To deal with missing data, we used sequential regression modelling to impute the missing values in the NEWS scales, QoL scores and covariates [39]. Twenty imputations were generated, taking into account 14% of missing cells in the original dataset. Descriptive statistics were estimated for all the NEWS subscales, HR-QoL components, and covariates with and without imputation. Then, we regressed mental and physical HR-QoL scores (in separate models) on the newly formed NEWS subscales, adjusting for covariates, with multiple imputed dataset. Generalized Estimating Equation (GEE) models [40] were used to account for the clustering effect of park neighborhoods. Sensitivity analyses were conducted using the complete cases for GEE modeling.

To test the multicollinearity between NEWS subscales, variance inflation factors (VIF) were calculated in the GEE models. The VIF for the NEWS subscales ranged from 1.1 to 1.6, showing no multicollinearity among NEWS subscales [41].

All analyses were performed using R version 4.0.4 [42]. The package of “psych” in R was utilized for the EFA [43]. We used the “MICE” package in R for multiple imputations [44]. The “geepack” package in R was utilized to test GEE models [45]. Alpha was set at *p* < 0.05.

## Results

### Factor analysis of NEWS subscales

Using the raw data, Cronbach’s alpha showed poor internal consistency for three of the original survey subscales: places for walking and cycling (0.57, 95% Confidence Interval (CI) = 0.54–0.61), safety from traffic (0.64, 95% CI = 0.61–0.67), safety from crime (0.66, 95% CI = 0.63–0.69). Neighborhood surroundings had good internal consistency with a Cronbach’s alpha of 0.79 (95% CI = 0.77–0.80) and neighborhood satisfaction had high internal consistency with a Cronbach’s alpha of 0.91 (95% CI = 0.90–0.92).

To adapt NEWS for NYC, EFA was performed for the above scales besides neighborhood satisfaction. EFA yielded two subscales: enablers (e.g., trails, sidewalks, esthetics) vs. barriers (e.g., high crime and traffic) of walkability. EFA results for the first two subscales with items demonstrating a factor loading ≥0.40 are shown in Table 1. Cronbach’s alpha was 0.84, 0.78 and 0.91 for walkability enablers, walkability barriers, and neighborhood satisfaction, indicating good internal consistency. The higher the NEWS subscale value, the stronger agreement on the statements for enablers and barriers.

**Table 1:** Exploratory factor analysis of the Neighborhood Environment Walkability Scale (NEWS)

Subscale	Items	Factor Loading		
NEWS walkability enablers^a^ (Cronbach’s α = 0.84)	The sidewalks in my neighborhood are well maintained (paved, even, and not a lot of cracks).	0.515		
	There are bicycle or pedestrian trails in or near my neighborhood that are easy to get to	0.467		
	There are trees along the streets in my neighborhood	0.445		
	Trees give shade for the sidewalks in my neighborhood	0.486		
	There are many interesting things to look at while walking in my neighborhood	0.607		
	My neighborhood is generally free from litter	0.563		
	There are many attractive natural sights in my neighborhood (such as landscaping, views)	0.586		
	There are attractive buildings/homes in my neighborhood	0.586		
	The speed of traffic on the street I live on is usually slow (30 mph or less)	0.433		
	The speed of traffic on most nearby streets is usually slow (30 mph or less)	0.451		
	There are crosswalks and pedestrian signals to help walkers cross busy streets in my neighborhood	0.445		
	The crosswalks in my neighborhood help walkers feel safe crossing busy streets	0.541		
	My neighborhood streets are well lit at night	0.557		
	Walkers and bikers on the streets in my neighborhood can be easily seen by people in their homes	0.473		
NEWS walkability barriers^a^ (Cronbach’s α = 0.78)	There is a high crime rate in my neighborhood		0.557	
	The crime rate in my neighborhood makes it unsafe to go on walks during the day		0.534	
	The crime rate in my neighborhood makes it unsafe to go on walks at night		0.625	
	There is so much traffic along the street I live on that it makes it difficult or unpleasant to walk in my neighborhood		0.525	
	There is so much traffic along nearby streets that it makes it difficult or unpleasant to walk in my neighborhood		0.524	
	Most drivers exceed the posted speed limits while driving in my neighborhood		0.424	
	When walking in my neighborhood, there are a lot of exhaust fumes (such as from cars, buses)		0.479	
Neighborhood Satisfaction^b^ (Cronbach’s α = 0.91)	Highway access from your home			0.43
	Access to public transportation in your neighborhood			0.42
	Commuting time to work/school			0.51
	Access to shopping in your neighborhood			0.57
	Number of friends you have in your neighborhood			0.49
	Number of people you know in your neighborhood			0.49
	How easy and pleasant it is to walk in your neighborhood			0.76
	How easy and pleasant it is to bicycle in your neighborhood			0.68
	Quality of schools in your neighborhood			0.61
	Access to entertainment in your neighborhood (restaurants, movies, clubs, etc.)			0.65
	Safety from threat of crime in your neighborhood			0.70
	Safety from threat of violence (or violent crime) in your neighborhood			0.71
	Amount and speed of traffic in your neighborhood			0.60
	Noise from traffic in my neighborhood			0.56
	Number and quality of food stores in your neighborhood			0.63
	Number and quality of restaurants in your neighborhood			0.63
	Neighborhood as a good place to raise children			0.66
	Neighborhood as a good place to live			0.67

^a^Response were in 4 categories: 1 = Strongly Disagree, 2 = Somewhat disagree, 3 = Somewhat agree, 4 = Strongly agree

^b^Response were in 5 categories: 1 = Strongly dissatisfied, 2 = Somewhat dissatisfied, 3 = Neither satisfied nor dissatisfied, 4 = Somewhat satisfied, 5 = Strongly satisfied

### Participant characteristics and bivariate associations of NEWS and HR-QoL

Table 2 provides the descriptive statistics of the sample with and without imputation. The imputed results closely matched those of the non-imputed data. The sample had a mean (±standard error) age of 38.8 ± 12.5 years and was 80.1% female. The vast majority of the participants were Latino (54.1%) or Black (34.6%). Just over half of the participants were in a household with an annual income lower than $20,000 while almost half had some college or a higher degree. In terms of employment, 37.5% of the sample was employed and 11.4% was self-employed). Just over one-third of the participants were married or living with a partner. Table 3 shows significant crude associations between NEWS scales and HR-QoL. The correlation coefficients of NEWS walkability enablers, NEWS walkability barriers, and NEWS neighborhood satisfaction with physical HR-QoL are 0.086, − 0.151 and 0.098 (*p* < 0.05). The correlation coefficients of NEWS walkability enablers, NEWS walkability barriers, and NEWS neighborhood satisfaction with mental HR-QoL are 0.180, − 0.143 and 0.218 (*p* < 0.05).

**Table 2:** Descriptive statistics

	Without MI		With MI	
	N	%	N	%
Gender				
Male	249	19.9	249	19.9
Female	1000	80.1	1003	80.1
BMI				
Under and Normal (< 25 kg/m^2^)	308	26.1	330	26.4
Overweight (25–29.9 kg/m^2^)	378	32.0	399	31.9
Obese (≥30 kg/m^2^)	495	41.9	523	41.8
Income				
$20,000 or more	496	45.4	566	45.2
Less than $20,000	596	54.6	686	54.8
Education				
Less than HS	226	18.7	234	18.7
HS graduate	405	33.4	418	34.6
Some college or college graduate	580	47.9	600	47.9
Employment				
Employed	455	37.9	469	37.5
Self-employed	133	11.1	143	11.4
Homemaker	137	11.4	144	11.5
Student	90	7.5	92	7.3
Retired	63	5.3	66	5.3
Unable to work	131	10.9	136	10.9
Unemployed for 1 year or more	81	6.8	84	6.7
Unemployed for less than 1 year	72	6.0	78	6.2
Don’t know/not sure	38	3.2	40	3.2
Marital status				
Married	333	27.3	340	27.2
Divorced	83	6.8	84	6.7
Widowed	34	2.8	37	3
Separated	120	9.9	123	9.8
Never married	537	44.1	553	44.2
A member of an unmarried couple living together	111	9.1	115	9.2
Ethnicity				
Hispanic	563	53.9	678	54.1
Black	372	35.6	433	34.6
Other	110	10.5	141	11.3
	Mean	SD	Mean	SD
Age	38.8	12.5	38.8	12.5
NEWS walkability enablers (range = 1–4)	2.7	0.5	2.7	0.5
NEWS walkability barriers (range = 1–4)	2.6	0.6	2.6	0.6
NEWS neighborhood satisfaction (range = 1–5)	3.4	0.8	3.4	0.8
Physical HR-QoL (0–100)	46.0	9.7	45.8	9.5
Mental HR-QoL (0–100)	48.7	11.3	48.9	11.1

*NEWS* Neighborhood Environmental Walkability Survey, *HR-QoL* health-related quality of life

**Table 3:** Spearman correlation of NEWS subscale and HR-QoL

	Mental HR-QoL	Physical HR-QoL
NEWS walkability enablers	0.180^*^	0.086^*^
NEWS walkability barriers	−0.143^*^	−0.151^*^
NEWS neighborhood satisfaction	0.218^*^	0.098^*^

^*^p-value < 0.05

### Regression analysis

Using separate GEE models with multiple imputations, mental and physical HR- QoL scores were regressed on NEWS subscales adjusting for covariates (Table 4). Results (with multiple imputations) showed mental HR-QoL was negatively associated with barriers of walkability (β ± SE = − 1.63 ± 0.55, *p* = 0.003) but positively associated with neighborhood satisfaction (β ± SE = 1.55 ± 0.66, *p* = 0.02), after adjusting for covariates. Physical HR-QoL was associated with only barriers of walkability (β ± SE = − 1.13 ± 0.57, *p* < 0.05). Sensitivity analyses showed the regression results were similar in imputed vs. non-imputed data (data not shown).

**Table 4:** GEE model results for mental and physical health-related quality of life (HR-QoL)

	Outcome variable					
	Mental HR-QoL			Physical HR-QoL		
	With MI			With MI		
	β	SE	*p*	β	SE	*p*
NEWS walkability barriers	−1.63	0.55	0.003	−1.13	0.57	0.05
NEWS walkability enablers	1.55	0.88	0.08	0.71	0.71	0.32
NEWS neighborhood satisfaction	1.55	0.655	0.02	0.24	0.51	0.64

Adjusted for age, BMI (underweight and normal < 25; overweight 25–29.9; obese > = 30), gender (male or female), income ($20,000 or more or less than $20,000), education (less than HS; HS graduate; some college or college graduate; employed; self-employed; homemaker; student; retired; unable to work; unemployed for 1 year or more; unemployed for less than 1 year; don’t know/not sure), marital status (married; divorced; widowed; separated; never married; a member of an unmarried couple living together), and ethnicity (Latino, Black, other)

*SE* standard error, *MI* multiple imputation

## Discussion

This is one of the first studies to examine HR-QoL in relation to perceived neighborhood environment. In particular, our study adds to the emerging literature with a specific focus on Latino and Black residents in lower income neighborhoods. We found that high perceived barriers of walkability was associated with both lower physical and mental HR-QoL. In addition, a global scale of neighborhood satisfaction was positively related to mental, but not physical, HR-QoL.

Despite the ubiquity of NEWS and SF-12 (or SF-36) in the literature, there have been surprisingly few studies investigating the relationship between these two sets of measures. In prior research, an Australian study on people aged 75 or greater showed a positive correlation between the original NEWS subscales and physical and mental HR-QoL using SF-36, but the study did not consider covariates [26]. Another study conducted in adults of different ages in 6 urban centers of China used multivariable models to examine each original NEWS subscale in relation to mental and physical HR-QoL (measured by SF-12) and found that higher perceived land use diversity, safety and esthetics were associated with higher physical and mental well-being [27]. Our study is the first to examine the association of the NEWS in relation to HR-QoL in the United States, especially among minority populations.

HR-QoL is an important public health outcome given the growing body of literature showing it to be an independent predictor of diverse clinical outcomes [46–54]. Physical HR-QoL as measured by SF-12 or SF-36 has been associated with the mortality of patients with hemodialysis [48], after coronary artery bypass graft surgery [54], and within 48 hours of admission to the ICU [52], as well as the development of obesity [55], diabetes [56], cardiovascular disease [57], and several cancers (e.g., oral [53] and advanced breast cancer [51]). In addition, patients with better mental HR-QoL scores were shown to be more likely to improve after lumbar fusion [49]. Mental HR-QoL has also been associated with mental health outcomes, such as anxiety [58], depression [59], and relapse of schizophrenia at 24-month follow up [47]. Therefore, HR-QoL can be considered a proxy of global well-being but more research is warranted on how to intervene on HR-QoL, including potentially via environmental strategies such as improving neighborhood walkability.

The items for the barriers for walkability subscale were a function of perceived traffic and crime. Our results are corroborated by prior research linking these environmental factors to health. For example, a previous study on children found that children who were exposed to high traffic volumes had significantly higher odds of asthma [60]. In addition, exposure to traffic congestion has been associated with on-the-job elevations of urinary catecholamines (a marker of stress) among bus drivers [61]. Elsewhere, the safer or less crime individuals feel in their neighborhood, the better mental health outcomes they have such as lower distress [62, 63] and better physical health [64]. Collectively, these and our findings point to the potential importance of transportation and neighborhood design in urban areas where the alleviation of traffic [65] and improvement in perceived safety [62, 66–68] may contribute to population well-being, which in turn may have a downstream impact on reducing health disparities.

It was notable that neighborhood satisfaction was significantly associated with mental but not physical HR-QoL. Neighborhood satisfaction has previously been found to be a predictor of mental health outcomes [69]. Neighborhood satisfaction has also been studied as a significant mediator between the quality of green space in a neighborhood and general health outcomes [18]. It is possible that mental HR-QoL serves as a mediator between neighborhood satisfaction and physical HR-QoL over time; future longitudinal studies are needed to further examine this hypothesis.

This study highlights a caveat for the generalizability of well published psychosocial scales such as NEWS without further psychometric testing. NEWS has been widely used in research globally [35, 70–72]. However, items in NEWS may be context-specific and may need to be customized to specific study populations [26, 70]. An important contribution of this paper is the application of EFA to reconstruct the factors, an approach that could be considered for future studies using surveys of perceptions of the environment.

This study and its findings add to the literature on urban livability. Urban livability is a multifaceted concept that incorporates diverse aspects of the neighborhood environment, including physical, biological and socioeconomic characteristics and their interactions [73, 74]. HR-QoL in this literature may be conceptualized, for instance, in terms of the number or density of health-related facilities and services [73]. Our study shows that the lived experience of community residents is an additional important dimension to consider. As such, this study has important research and policy implications that require the convergence of public health, urban planning and design, and other fields in a more holistic approach to urban livability.

Several limitations are inherent in this study. The study was cross-sectional; thus, no causality could be inferred. The target population included mostly residents of low-income, minority neighborhoods, limiting the generalizability of our findings to all of NYC or elsewhere. In addition, we cannot rule out the possibility of selection bias among those who chose to participate in the survey study, thus study findings may not be representative of the entirety of the underlying communities. The population focus, however, was also a strength of the study given the heightened health disparities experienced by Latino and Black communities in the United States.

In conclusion, perceptions of the built environment appear to be important factors in the HR-QoL of low-income residents in NYC. Further research is warranted to investigate the pathways by which such perceptions influence HR-QoL, including potential stress mechanisms. The current paper adds to the literature on urban health and urban planning and shows the potential value of incorporating community members’ experiences of the built environment and robust HR-QoL measures in studies of population well-being and environmental livability.

## Data Availability

The datasets generated and/or analyzed during the current study are not publicly available due to the fact that this is an ongoing study but are available from the corresponding author on reasonable request.
